# Pixel Selection by Successive Projections Algorithm Method in Multivariate Image Analysis for a QSAR Study of Antimicrobial Activity for Cephalosporins and Design New Cephalosporins

**Published:** 2018

**Authors:** Ahmadreza Amraei, Ali Niazi, Mohammad Alimmoradi, Bahram Delfan

**Affiliations:** a *Department of Chemistry, Arak Branch, Islamic Azad University, Arak, Iran.*; b *Department of Chemistry, Central Tehran Branch, Islamic Azad University, Tehran, Iran. *; c *Lorestan University of Medical Sciences, Department of Physiology and Pharmacology Khorramabad, Iran.*

**Keywords:** Cephalosporin, QSAR, Partial least squares, Multivariate image analysis, Successive projections algorithm, Pixels of selection, Acid dissociation constant

## Abstract

Thirty-one Cephalosporin compounds were modeled using the multivariate image analysis and applied to the quantitative structure activity relationship (MIA-QSAR) approach. The acid dissociation constants (pK_a_) of cephalosporins play a fundamental role in the mechanism of activity of cephalosporins. The antimicrobial activity of cephalosporins was related to their first pK_a _by different models. Bidimensional molecular structures (images) were used to calculate some pixel descriptors. The selection of pixels by successive projections algorithm (SPA) was done to achieve simple MIA-QSAR models; based on a small subset of pixels. In the present study, the performance of pixel selection technique using SPA for partial least squares (PLS) model was evaluated. The obtained model showed nice prediction ability with root mean square error of prediction (RMSEP) values of 0.402, 0.315, and 0.160 for principal component regression (PCR), PLS and SPA-PLS models respectively. Among the three methods, SPA-PLS was potentially useful in predicting the pK_a_ of cephalosporins. The study showed the maximum structural efficacy is on pK_a_ in a, b and c regions.

## Introduction

Cephalosporins (Cephems) are broad and they are chemically related to the β-lactam class of antibiotics with enormous medicinal applications (1). Cephalosporins exhibit good antibacterial properties against a broad class of bacteria including Gram-positive and Gram-negative bacteria (2). Cephalosporins such as penicillin can prevent bacterial cell wall synthesis. The properties such as antimicrobial activity, chemical stability, solubility, and acid-base properties depend on enormous extent on cephalosporin structures (1). The human body›s resistance against antibiotics is a major problem in the medical community; so, it is expedient that new cephalosporins be designed (3). The pK_a_ play a fundamental role in the mechanism of activity of various biological fluids, primarily the blood, and its capability to interact with components of these fluids and other drugs can be investigated (1). The activities applied in models QSAR contain biological activity, chemical measurement, toxicity and bioavailability and are used as dependent variable in building a model (4-6). In order together useful information for medicinal chemistry, design of new drugs and toxicity, QSAR is one of the well-established key areas in chemometrics. The QSAR models were created with successful prediction of the activity and factors influencing the activity, and were used at the end to design compounds that were more effective (7-11). The steps necessary in obtaining a MIA-QSAR model include drawing molecular structures, molecular descriptors (pixels) calculation, splitting of data for training and validation sets, pixels selection, model build-up between selected variables and activity, and finally model validation (8). Due to the large number of descriptors in MIA-QSAR, a major step in constructing the model is the selection of a subset of pixels to maximize information contents. Variable selection techniques in MIA-QSAR (12-17) play a key role in developing work of this nature because of the high dimensional data sets. Multivariate calibration model such as PCR and PLS is a technique that can be effective in dealing with the problem of undesirable increase in variable/object ratio and collinearity (18). The SPA is a forward selection method that starts with one variable; and incorporates a new one during each iteration until a specified number (N) of variables is obtained. Previous studies have shown that SPA can be used successfully as a special variable selection method (19-23). In recent years, many applications for image analysis have been created to solve a variety of problems due to rapid low costanalysis. Image analysis is a wide field of study that encloses classical studies on gray scale or (red-green-blue) RGB images. Esbensen and Geladi have demonstrated that multiple image analysis may provide useful information in chemistry; the descriptors do not have a direct physicochemical meaning since they are binaries (24). In MIA-QSAR (25-27) bidimensional images have been shown to contain chemical information that allows the relationship between chemical structures and activities. In this study, emphasis was on the application of 2D images, which are the suitable structures of compounds that can be drawn with the help of any appropriate software, pixels images as descriptors in QSAR (28, 29).The obtained MIA-QSAR model was then tested with successful prediction of the pK_a_ of 4 cephalosporin compounds.

## Experimental

*Hardware and Software*

The SONY Personal Computer (4 GB RAM) equipped with Windows 8 operating system and MATLAB (Version 7.8.0 (R2009), Math work Inc.) was used. The PLS calculus were carried out by using the PLS-Toolbox (Version 4.0) (Eigenvector Technologies). SPA program was written in MATLAB by M.C.U. Araujo *et al*. The source codes of the programs were made available by the authors upon our request and molecular structures were drawn by the use of Chem Office software (Version 2010). Kennard-Stones programs were written in MATLAB according to the algorithm (30).

*Data Set*

The first pK_a_ of 31 cephalosporins were taken from the article of VG Alekseev (1). The chemical structure of these cephalosporins and their corresponding first pK_a_ data are listed in Table1. In order to create a reliable MIA-QSAR model, data set was separated into the parts of training and prediction sets according to the Kennard-Stone algorithm. Kennard-Stone algorithm is one of the ideal ways of splitting a set of known data. The Kennard–Stone algorithm selects a set of molecules in studied set of data, which are uniforhy distributed over the space defined by the candidates. This is a classic technique to extract a representative set of molecules from a given data set. In this technique the molecules are selected consecutively. The first two objects are chosen by selecting the two farthest apart from each other. The third sample chosen is the one farthest from the first two objects, etc (31).

**Table 1:** Chemical structures of cephalosporins and their corresponding pKa

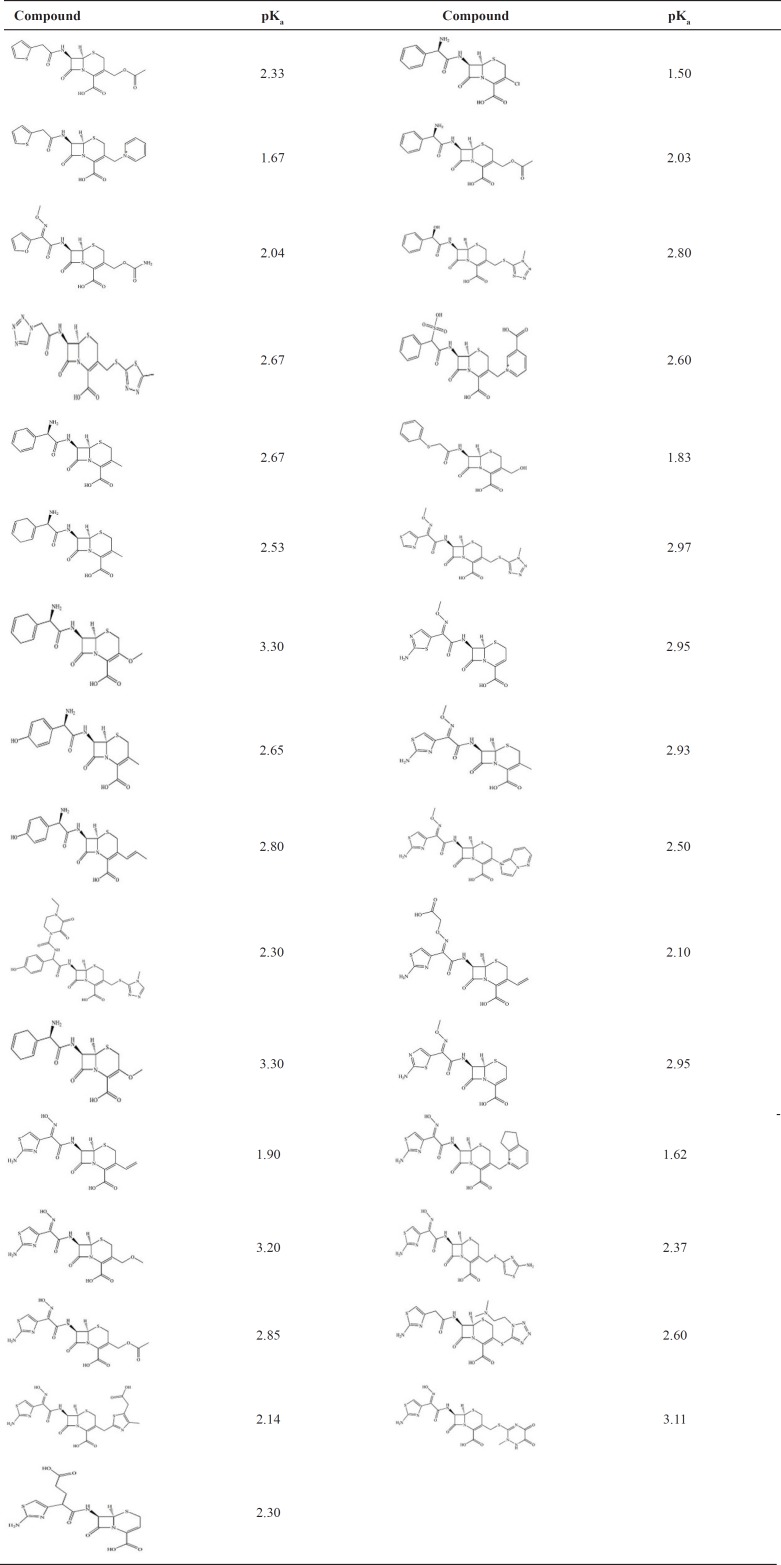

*Multivariate Image Analysis Descriptors*

The descriptors in the MIA-QSAR method are the pixels of structure images. The pixels chosen by SPA were correlated with the dependent variables for building the MIA-QSAR model. The bi-dimensional structures of each compound in Table 1 were systematically drawn using the Chem Office software and then converted to bitmaps of 240 × 160 pixels workspace. All the cephalosporin structures were fixed accordingly; a pixel was fixed on the sulfur element in the 110 × 80 pixel coordinate since the whole images must be superimposed afterwards to obtain maximum similarity. Each structure had a 2D image; superposition (alignment) of the 31 images gives a three-way array of 31× 240 × 160 dimension, which was unfolded to a two-way array (matrix) of 31 × 38400 dimension. Many columns with zero variance were removed. 

## Results and Discussion

*Multivariate Image Analysis Descriptors*

**Figure 1:**
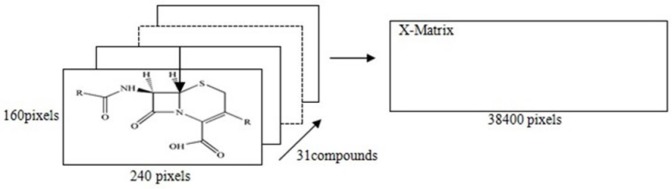
2D images and unfolding step of the 31 chemical structures to give the X-matrix. The arrow in structure indicated the coordinate of a pixel in common among the whole series of compounds, used in the 2D alignment step

The MIA-QSAR model was made based on the correlation of these pixels with the activities of cephalosporin. The bi-dimensional structures of each cephalosporin in Table 1, were drawn using the Chem Office software as well as the same font and size, and thereafter, converted to bitmaps of 240 × 160 pixels workspace. All built molecular structures were systematically fixed in a given coordinate. In this study, the point fixed at the 110 × 80 coordinate (sulphur element) was used as reference in the alignment step as illustrated in Figure.1. Since each molecular image is a bi-dimensional image, alignment of the 31 images gives a three-way array of 31 × 240 × 160 pixels which was unfolded into 38400 rows and then the 31 images were grouped to form 31 × 38400 matrix. In order to minimize the memory, the columns with zero variance were eliminated, and at the end all pixels data were mean centered.

*Principal Component Analysis of the Data Set*

**Figure 2:**
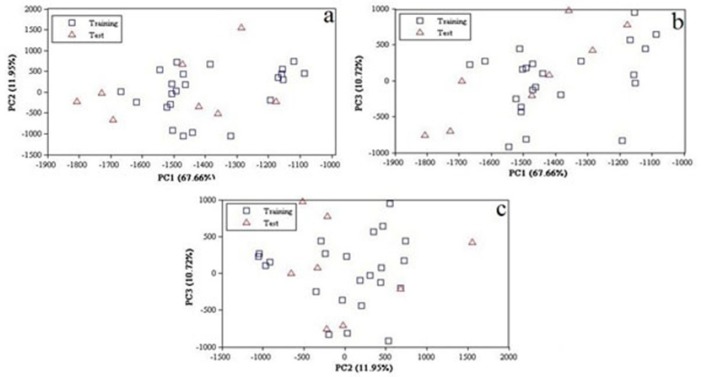
Principal components of the 2D image descriptors for the data set, (a), PC1 versus PC2, (b) PC1 versus PC3 and (c) PC2 versus PC3

The number of real dimensions of data sets was determined using PCA. It is also used for making 2, 3 dimensional plots of data for visual examination in order to diagnose collinearity, homogeneities in the data set and reduce high dimension as well as interpretation for detection of outliers and identification of clusters. PCA were performed within the calculated image descriptors space for the whole data set. The number of principal components is less than or equal to the number of original variables. The first PCs retained the greatest amount of variation and valuable information in the data set. A total of 248 pixel descriptors were initially calculated using PCA for the entire data set of 31compounds. The PCA results showed that three PCs (PC1, PC2 and PC3) had a value of 90.56% of the overall variability: PC1 = 67.66%, PC2 = 11.95% and PC3 = 10.72% (Figure.2) and thus the images domain may be expressed in terms of these 3 dimensions mainly. Since almost all variables can be accounted for by the three primary PCs, their score plot is a reliable tool for the spatial distribution of the points of the data set. As shown in Figure 2, there is no obvious clustering among the compounds. Good data distribution on the development of reliable and robust QSAR models is very important. The ability and qualityof the prediction depends on the data set used to build the model. In order to modeling, data set were divided into two groups; training set (23 data) and a prediction set (8 data) according to Kennard-Stones­ algorithm. According to Figure 2, the distribution of compounds in each subset of pixels appears somewhat well diffused over the space of the principal components.

*PCR and PLS Modeling*

**Figure 3:**
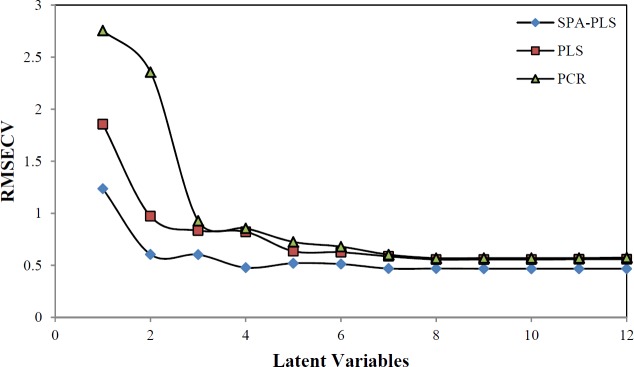
The RMSECV versus number of latent variables

The main step in the MIA-QSAR method is the relationship between several pixels and dependent variables. The PLS and PCR methods were used as the multivariate calibration methods. The root mean square error cross validation (RMSECV) was applied in finding the number of appropriate latent variables required for description of the best developed model (Figure 3). The F-statistical test was used to determine the significance of RMSECV values that are greater than the minimum. The values of RMSECV were minimal when the optimum value of latent variables (LVs) were 7, 6 and 4 for PCR, PLS and SPA-PLS, thus the optimum number of LVs for the training set of SPA-PLS method was chosen to be 4. Before modeling, the data set were mean centered.

*SPA-PLS Modeling*

**Figure 4:**
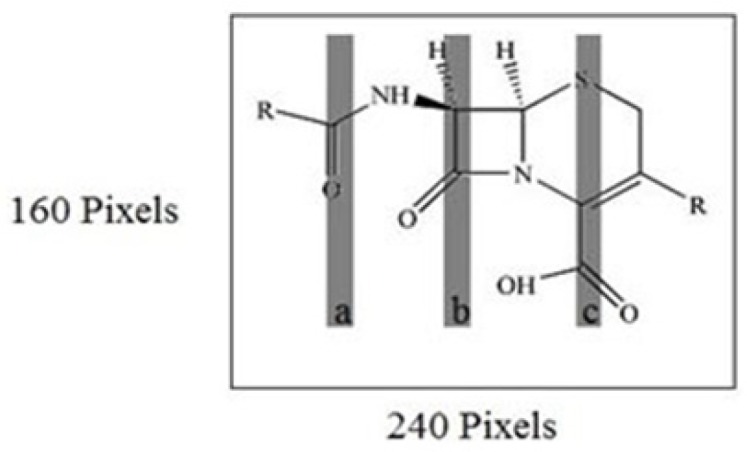
Selected regions by successive projection algorithm

As you know, one of the key problems in a modeling that is robust and fast is the selection of subset of molecular descriptors (pixels) instead of all set descriptors. SPA is a forward variable selection method. In order to obtain subsets of data set with small collinearity, employ simple operation in vector space of variables (pixels). Data sets were mean centered before the SPA-PLS was performed. After pixels selection by SPA technique, these pixels were used for running the PLS. By running the SPA-PLS, the number of latent variables reduced to 4 (Figure 3). The selected areas by SPA shown are in Figure.4. In this work, it has been shown that SPA can be a good, reliable and fast technique for pixel selection in MIA-QSAR. Using the chosen pixels by SPA, it was found that the most structure influence was on the pK_a_ in a, b and c regions (Figure.4) and among these three regions, b in which there were more alterations by different functional groups had more influence on pK_a_ than in other regions.

*Model Validation and Prediction of pKa*

In Table 2, the predicted values of pK_a _calculated by the PCR, PLS and SPA-PLS methods and the percent relative errors of prediction are presented. The correlation between reference and predicted pK_a_ for SPA-PLS was suitable with R^2 ^=.9351 and intercept = 5576. The statistical results presented in Table 2 clearly indicate that the SPA-PLS model has good quality with low prediction errors. In addition, to evaluate the predictive ability of a different model, the root mean square error of prediction (RMSEP), relative standard error of prediction (RSEP) and cross validated coefficient (Q^2^) can be used.

$\mathrm{RMSEP}=\sqrt[{2}]{\frac{\sum_{i=1}^{n}{\left(y_{\mathrm{pred}}-y_{\mathrm{obs}}\right)}^{2}}{n}}$

 Equ 1.

$\mathrm{RSEP}(\%)=100\times \sqrt[{2}]{\frac{\sum_{i=1}^{n}{\left(y_{\mathrm{pred}}-y_{\mathrm{obs}}\right)}^{2}}{\sum {\left(y_{\mathrm{obs}}\right)}^{2}}}$

Equ 2.

Where y_pred_ and y_obs_ is the predicted value and observed value of the sample and n is the number of samples in the validation set. These statistical parameters with their goodness qualities are displayed in Table 3.

**Table 2:** **Observation and calculation values of pK**_a_ **using PCR, PLS and SPA-PLS models**

**Number of compounds (** Table 1 **)**	**Observation pK**_a_	**PCR**		**PLS**		**SPA-PLS**	
		**Predicted**	**Error (%)**	**Predicted**	**Error (%)**	**Predicted**	**Error (%)**
8	2.60	2.36	-10.17	2.51	-3.58	2.56	-1.56
24	1.62	1.97	17.76	1.90	14.73	1.83	11.47
27	2.85	2.51	-13.54	2.54	-12.20	2.81	-1.42
20	2.10	2.58	18.60	2.50	16.00	2.21	4.97
21	2.91	2.34	-24.35	2.53	-15.01	2.57	-13.22
25	3.20	2.60	-23.07	2.67	-19.85	3.07	-4.23
18	2.50	2.34	-6.83	2.6	3.84	2.42	-3.30
19	2.03	1.79	-13.40	2.17	6.45	1.97	-3.04

**Table 3:** **Comparison of the statistical parameters by different QSAR models for the prediction of the pK**_a_

**Methods**	**PCR**	**PLS**	**SPA-PLS**
NLV^a^	7	6	4
RMSEP	0.402	0.3157	0.160
REP (%)	25.62	20.11	10.11
R^2^	0.4629	0.7375	0.9351
Q^2^	0.3340	0.5900	0.8960

^a^ Number of latent variableQ^2^Coefficient for the model validation by leave-one-out.

*Molecular Design*

As a created application method, we investigated SPA-PLS models to predict the pK_a_ of 4 new cephalosporin compounds whose biological tests have not been performed with this application method. In Table 4, the chemical structure of 4 new cephalosporins compounds and their pK_a _calculated by this proposed method are presented.

**Table 4:** **Structural modification of cephalosporins and predicted pk**_a_ **by SPA-PLS**

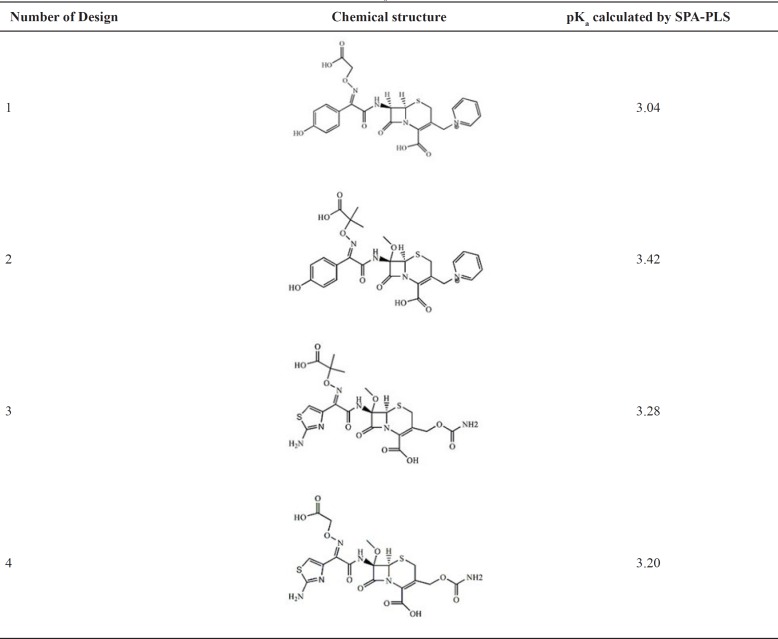

## Conclusion

In this work, using the SPA-PLS model, a QSAR model that was useful in the prediction of the pK_a_ of 31 cephalosporins based multivariate image analysis alone have been proposed. In addition, the SPA-PLS model showed nice and accurate predictive values giving good correlation values in calibration. Pixels selection improved the predictive quality of the MIA-QSAR model. Pixel selection using the SPA method unlike other methods appears to be fast and reproducible. Our study showed that pixels selection by the SPA method is useful for those designing novel cephalosporins.
